# A Slaughterhouse-Linked Spatial Framework for Prioritizing Livestock Surveillance in Thailand

**DOI:** 10.3390/ani16121767

**Published:** 2026-06-08

**Authors:** Pongpon Homkong, Veerasak Punyapornwithaya, Warangkhana Chaisowwong, Napat Harnpornchai, Terdsak Yano

**Affiliations:** 1 Faculty of Veterinary Medicine, Chiang Mai University, Chiang Mai 50100, Thailandwarangkhana.chai@cmu.ac.th (W.C.); 2 Veterinary Service, Department of Public Health and Environmental, Chiang Mai Municipality, Chiang Mai 50200, Thailand; 3 Research Center for Veterinary Biosciences and Veterinary Public Health, Faculty of Veterinary Medicine, Chiang Mai University, Chiang Mai 50100, Thailand; pveerasak.r@gmail.com; 4 Faculty of Economics, Chiang Mai University, Chiang Mai 50200, Thailand; napaticdi@gmail.com

**Keywords:** animal health surveillance, veterinary epidemiology, slaughterhouse surveillance, spatial prioritization, livestock systems, One Health, Thailand

## Abstract

(1) Background: Slaughterhouses receive livestock from different sources, enabling the collection of health-related surveillance information. Few surveillance frameworks integrate slaughterhouse distribution with livestock population density for prioritizing surveillance areas. (2) Methods: The surveillance priority of livestock in Thailand was determined on a district basis. Registered slaughterhouses were mapped using kernel density estimation. Livestock were mapped using census data. The z-scores were combined to determine the composite surveillance prioritization index for each district. The 90th percentile was used to determine district-level surveillance priority. Spatial analyses using Global Moran’s I and LISA statistics were performed. Sensitivity analyses assessed robustness. (3) Results: The composite district-level priority index ranged from −0.728 to 9.071 (I = 0.642, *p* < 0.001). LISA identified 120 High–High (HH) priority districts. Of these, 42 priority districts were selected, concentrated in the northeastern and west-central regions. (4) Conclusions: This model is replicable and can be applied to other regions that rear livestock and use slaughterhouses for surveillance.

## Simple Summary

Slaughterhouses are critical points for livestock health surveillance. These locations are useful for monitoring livestock health conditions in their surrounding regions and supporting early surveillance planning. It is, however, exceedingly challenging to determine the precise areas that should be prioritized for surveillance at the national level. To address this issue, data regarding the number of animals in each district of Thailand, as well as the locations of registered slaughterhouses within the country, were collected. The analysis revealed that priority districts were mainly located in the northeastern and west-central regions of the nation, encompassing 42 districts in total. These districts were further grouped into seven proposed operational zones for field planning. Overall, this framework will allow the veterinary authorities of Thailand to more effectively allocate surveillance resources for livestock health monitoring and inspection prioritization. Furthermore, the creation and implementation of this framework may support a more cost-effective and systematic approach to protecting animal health and public health nationwide. This study did not collect any data on disease occurrence, pathogen detection, animal movement, slaughterhouse capacity, or biosecurity, so the results from this study should be interpreted as a prioritization for surveillance and not as an actual prediction of disease risk.

## 1. Introduction

The livestock sector has rapidly intensified and become an integral part of complex supply chains worldwide, a structural evolution observed in numerous national and international settings [1]. While economic benefits may be perceived, increased potential for transboundary and emerging infectious diseases, such as foot-and-mouth disease (FMD), with downstream consequences for trade, rural livelihoods and food security, are immediately concerning. Within intensified systems, the slaughterhouse is an epidemiologically relevant interface as a converging point for animals from various herds; it may increase contact opportunities via transport, lairage and processing, and at the same time, it is a high-value surveillance-capable node for upstream control via meat inspection and throughput evaluation [2]. For example, in Thailand, interdisciplinary assessments have explored livestock movement networks which are, ultimately, slaughterhouse-oriented, establishing slaughterhouses as important nodes of information and transmission [3,4,5]. Thus, the location of these systems and other related slaughterhouses contributes to surveillance feasibility along certain livestock movement corridors [6]. In this scenario, the position of the slaughterhouse will be found where intensive livestock farming occurs, and livestock movement corridors will concentrate on high-throughput slaughterhouses as the main slaughtering points in the supply chain.

Spatial epidemiology renders a detailed toolkit for uncovering clustering, spatial gradients, and movement/interface-related feasibility that may go undetected in routine reporting [7]. Kernel density estimation (KDE) is widely used for mapping point-based occurrences (e.g., disease reports, markets, slaughterhouses) while Moran’s I (and Local Indicators of Spatial Association (LISA))—now commonly integrated into evidence-based spatial assessments for animal health governance—detects spatial autocorrelation and locates it [8]. Their application has been validated in the field in Thailand; for example, a spatial assessment surrounding the outbreak of lumpy skin disease (LSD) in 2021 afforded maps of disease-related spatial clusters supporting control [9]. Nationally and sub-nationally, farm distributions and livestock populations have been mapped to assess population-level risk factors and infrastructure dynamics relevant to control [6]. Yet a significant methodological and policy gap exists. Most studies assess infrastructure (e.g., markets or slaughterhouses per square km) or susceptible populations (e.g., livestock distribution) at a point in time. Evidence that combines slaughterhouse infrastructure density and population data to create an index that can be leveraged for policy based on spatial proximity is limited [10,11].

This gap limits the ability to identify where high-value surveillance infrastructure and livestock populations relevant to surveillance planning spatially overlap, which is essential for evidence-based surveillance planning. This study fills that gap by creating and utilizing a systematic approach to spatial analysis on a national scale across Thailand. For the slaughterhouse-oriented livestock movement network in Thailand, this is not merely a methodological convenience, but a useful basis for surveillance prioritization on a national scale [5]. Decisions made throughout the analysis conformed to standardized spatial-epidemiology practices and recognized processes from international surveillance guidance [4,12]. KDE serves as a continuous proxy for slaughterhouses based on point patterns but acknowledges spatial heterogeneity; livestock density surfaces provide population context for interpretation; Moran’s *I*/LISA provides inferential value to determinants of significance for clustering. Furthermore, we assess robustness through parametric sensitivity (e.g., kernel bandwidth, *k*-nearest neighbors, layering weights) and bootstrap resampling, ensuring that policy-relevant conclusions are not artifacts of tuning decisions. Therefore, the final surveillance prioritization index serves as an interpretable and scalable product from One Health-relevant spatial surveillance planning that can be re-estimated when new data become available or if infrastructure/population dynamics shift. Lastly, this work builds on existing decision-making frameworks in Thailand that relate spatial data to an operationalized control [13] but extends the work by incorporating slaughterhouse infrastructure and livestock layers into one reproducible spatial framework. The aim of this study was to develop a nationwide scale spatial framework for prioritizing livestock surveillance in Thailand based on the livestock density in the districts and density of registered slaughterhouses. In particular, the objectives were to (i) develop standardized spatial surfaces of slaughterhouse intensity and livestock density, (ii) build a composite slaughterhouse–livestock surveillance prioritization index based on the spatial superposition of slaughterhouses and livestock, and (iii) determine prioritized districts and proposed operational zones for allocation of surveillance resources. The framework will be used to inform surveillance planning and resource allocation. It should not be taken as an actual measure of the occurrence or transmission risk of disease as no information was available on disease incidence or pathogen detection, animal movement, slaughterhouse capacity, or biosecurity.

## 2. Materials and Methods

### 2.1. Study Area and Data Sources

A national-level spatial assessment of the 928 districts of Thailand was conducted. Two geodatabases were established.

**Legally registered slaughterhouses.** The Department of Livestock Development (DLD), Thailand, provided a point-feature shapefile of legally registered slaughterhouses available for the 2025 analytical framework. Registration indicates inclusion in the official DLD slaughterhouse database and was used to represent the formal slaughterhouse sector. Each registration has a unique number, spatial coordinates (WGS84), and administrative information (province, district).

**Livestock counts.** The total livestock count at the district level has been obtained from the national livestock census (2025 administrative boundary level), which includes beef cattle, dairy cattle, and buffalo. Counts at the species level were aggregated to the district level and then merged with the administrative polygons of the 928 districts for spatial analysis. Each polygon had an FID of a district representing the district within a province.

**Quality control.** Before any analyses, (i) duplicates were deleted, (ii) points outside of national borders were eliminated, and (iii) administrative codes were homogenized to one standardized national registration code for effective spatial joins. Missing livestock census values were treated as zero for calculation.

### 2.2. Analytical Design and Spatial Framework

All layers were reprojected into a common projected metric coordinate reference system for area and distance measurement across the Thai region (**EPSG:32647; WGS 84/UTM zone 47N**). All spatial surfaces were co-registered following a common analytical framework, a 5 km raster grid that spanned the country. The choice of grid resolution was made to strike a balance between district-level interpretations and national policy application, while at the same time offering a level of resolution that is practical.

### 2.3. Construction of Surveillance Priority Surfaces

Two continuous surfaces were created: livestock population density and slaughterhouse intensity.

**Livestock density surface**, *A(x,y)*. The district-level totals were converted into areal density (animals/km^2^) and rasterized to the 5 km grid where a uniform within-district distribution is assumed based on the lack of sub-district data; this is a standard assumption when generalizing national assessments. However, this assumption was assessed through sensitivity analysis (Section 2.6).

**Slaughterhouse kernel density surface**, *S(x,y)*. The slaughterhouses were treated as a planar point pattern. A Gaussian kernel density estimation (KDE) was run using density.ppp from spatstat.explore with a border correction to adjust for slight contributions for points that fell into edge buffers to avoid under-representation [14]. A pragmatic local scale for slaughterhouse density estimation, consisting of 10 km, was chosen as a local screening scale to avoid over-smoothing regional patterns while capturing them. The 5 km and 20 km bandwidth scenarios were tested in the sensitivity analysis as this bandwidth is not a measured animal movement radius or disease-transmission distance.

### 2.4. Composite Surveillance Prioritization Index and District Aggregation

A GIS-based multi-criteria decision analysis (GIS-MCDA) approach was used to derive the district-level surveillance prioritization, which was further based on a weighted linear combination (WLC) as recommended in veterinary spatial prioritization and decision analysis in data-scarce regions [15]:

$$R(x,y)\;=\;\alpha \;Z_{S}(x,y)\;+\;(1-\alpha )\;Z_{A}(x,y)$$

The primary analysis used a neutral baseline weight of α = 0.5 that assumes equal contributions from both components when no empirical data on weighting are available [16]. Each district d was given a value of surveillance priority based on the 90th percentile of the values of the composite index cells in that district:

Rp90d =  Rx,y : x,y∈d percentile 90

to emphasize upper-tail intensity relevant for targeting.

**Justification of criteria.** Livestock density is consistently found to drive transmission opportunity [6] while slaughterhouses act as hubs in trade networks, like live bird markets for avian influenza, creating aggregation interfaces and proxying high-contact movement nodes [3,6].

### 2.5. Spatial Clustering and Priority-District Identification

District-level spatial clustering of the surveillance prioritization index was assessed using Global Moran’s I for national-level spatial autocorrelation [17]. The spatial clusters were detected with Local Indicators of Spatial Association (LISA) based on the local Moran statistics in sfdep [18] with significant HH spatial clusters identified. Neighborhoods for the baseline analysis were based on the Queen contiguity criterion, which considers contiguous neighborhoods to be those with boundaries and vertices in common. Districts which were not contiguous were included in the dataset but not calculated as neighbors. A total of 999 Monte Carlo simulations were performed to determine the level of statistical significance. To see how sensitive the results are to k, k-nearest neighbors (KNN) weights were also tested (with k ranging from 4 to 12) and are presented in the Results section.

Shortlisting rule, multi-criteria decision analysis (MCDA). Districts were considered priority surveillance districts if all three of the following conditions were met:

**1.** Significant **High–High** (HH) LISA classification (*p* < 0.05);

**2.** $R_{p90}$ within the **top 15%** nationally;

**3.** Total livestock population greater than or equal to the provincial 75th percentile [15].

The top 15% threshold was selected to capture the upper tail of the national surveillance prioritization distribution while retaining an operationally manageable number of districts for field planning. The provincial P75 livestock criterion was included to preserve local livestock relevance within each province and to avoid excluding districts that were important within their provincial context but did not have the highest absolute livestock counts nationally. These thresholds were therefore used as pragmatic surveillance planning criteria rather than biological or disease-risk thresholds.

In terms of operational zoning, the 42 verified priority districts were clustered using the k-means algorithm with k = 7, seed = 123, and cluster centers defined as district centroid coordinates for ease of field implementation and mobilization of resources for the project. To show the geographic coverage of each proposed area, convex hull envelopes were created for the operational-zone map.These zones are not analytically validated disease-risk zones and are meant as an aid to the implementation of coordinated surveillance.

### 2.6. Sensitivity and Uncertainty Analysis

Two methods were used to assess robustness.

**Parameter sensitivity.** Critical parameters were varied: raster grid resolution (2.5 km; 10 km), KDE bandwidth (5 km, 20 km), and WLC weight (0.3, 0.7). For each altered parameter, surfaces were recreated; $R_{p90}\;$ was recalculated, clustering was re-run, and the shortlist was generated.

**Bootstrap stability.** To estimate the percentage of iterations in which each district was a “HH” district, bootstrap resampling with 200 iterations was performed with replacement and a new pipeline was recreated in each iteration. To compare across runs, the overlap between any given shortlist and the original shortlist was assessed using the **Jaccard index** and to assess how well the shortlist remained stable under parameter variation.

### 2.7. Software and Reproducibility

All analyses were conducted in R 4.3.2 with version control maintained through renv package to support reproducibility., and a configuration file level for the project (config.yaml) centralized parameters across developmental actions for compliance with reproducibility standards mandated after significant developmental milestones. Key packages included spatial management packages (sf version 1.0.21, terra version 1.8.70, point pattern analysis)/KDE packages (spatstat.explore version 3.5.3/spatstat.geom version 3.6.0) and a GIS-based statistics package (sfdep version 0.2.5.), while generic data manipulation is covered through core tidyverse packages including dplyr version 1.1.4, readr version 2.1.5. Package version control accessed the renv lockfile and exported sessionInfo() post-development with fixed seeds for any randomized operations. All outputs (rasters, district tables, maps) for dissemination efforts were created through scripted workflows to provide efficient updates as data changed over time.

## 3. Results

### 3.1. Spatial Distribution of Slaughterhouses and Livestock Population

**Figure 1:**
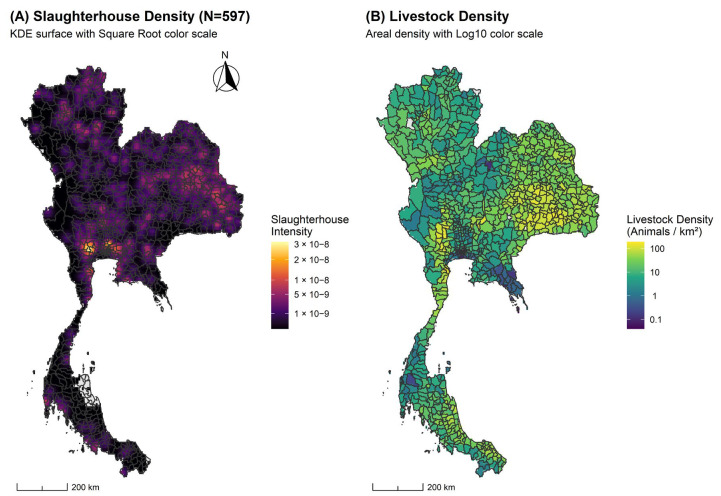
Spatial distribution of slaughterhouse density and livestock population in Thailand (2025, district level). (**A**) Kernel density estimation (KDE) surface from 597 certified slaughterhouses (Gaussian kernel, bandwidth of *h* = 10 km, border correction), projected with a square-root color scale to improve visualization of higher-density areas. (**B**) Livestock areal density (head/km^2^) from district census totals, projected with a log10 color scale to compensate for susceptibility to lower values. The two layers are projected in WGS 84 / UTM zone 47N (EPSG:32647) on a common 5 km grid; district border polygons are provided for clarity. Sources: Department of Livestock Development (locations of slaughterhouses) and official government livestock census (totals per district). Scale bar = 200 km.

A total of 597 officially regulated standardized slaughterhouses were recorded within the research area. Their spatial density via Gaussian KDE (*h* = 10 km) reveals large heterogeneities (Figure 1A) where slaughterhouses in proximity to one another exist in elevated concentrations across the central plains of Bangkok, throughout the entirety of the northeast and in select areas of overlap in the north and south.

Similarly, the population densities of livestock reveal similar levels of high heterogeneity (Figure 1B), where maximum populations exist in a central-western band across the country, with minor additional bands in more concentrated areas in the northeast and a more south up-and-down pattern along the peninsula. Therefore, the spatial co-distribution of slaughterhouse infrastructure and livestock populations is heterogeneous, suggesting that surveillance priority is geographically concentrated rather than uniformly distributed across the country.

### 3.2. National One Health Composite Surveillance Prioritization Surface

These two surfaces were z-score-standardized, combined with α = 0.5 between slaughterhouse intensity $S\left(x,y\right)\;$ and livestock density A(x,y) via a weighted linear combination, $R=\alpha Z_{S}+(1-\alpha )Z_{A}$ with α=0.5, with surveillance priority at the district level derived from the 90th percentile of cell values ($R_{p90}$) to capture an upper-tail intensity for appropriate focus. For the 928 districts combined, $R_{p90}$ fell between −0.728 and 9.071 (median 0.063) (Table 1).

**Figure 2:**
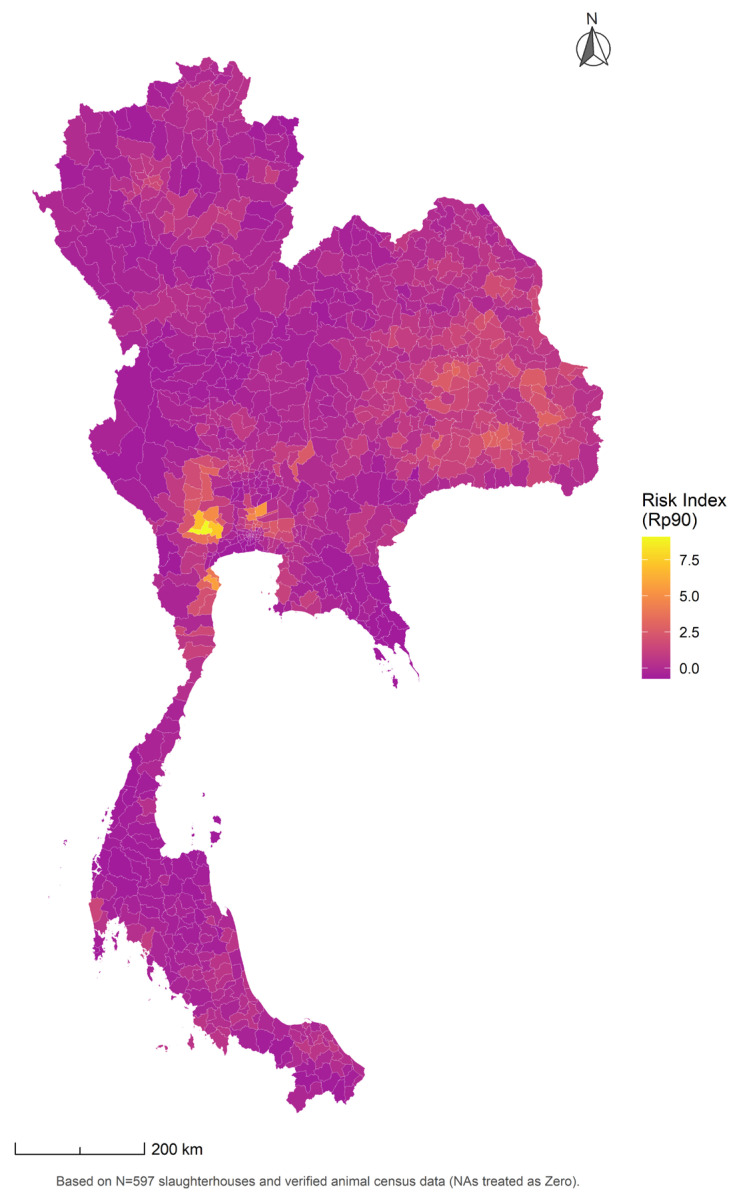
National composite surveillance prioritization surface for Thailand at the district level (*R*p90). District-level surveillance priority was summarized as the 90th percentile of the composite surface using equal weighting (α = 0.5) between standardized slaughterhouse intensity and livestock density. Warmer colors indicate areas with greater spatial overlap between registered slaughterhouse infrastructure and livestock populations. The analysis included 928 districts; missing livestock census values were treated as zero for calculation. CRS: WGS 84/UTM Zone 47N (EPSG:32647). Data sources: DLD-certified slaughterhouse data and the official 2025 livestock census.

The composite national surveillance prioritization map (Figure 2) combines these two districts to provide a relatively large, continuous high-priority zone extending through much of the northeast to the central plains and a second, smaller high-priority zone in the center/west; significantly, much of the north and much of the south maintain consistently lower associated values.

### 3.3. Spatial Clustering of High-Priority Districts

**Figure 3:**
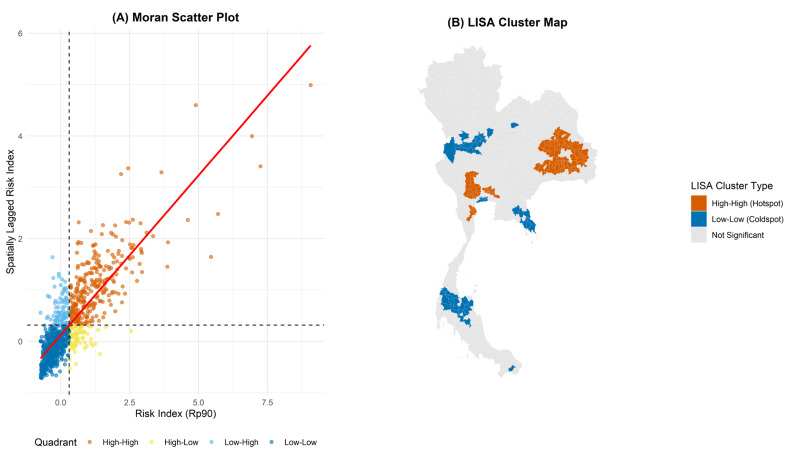
Spatial autocorrelation of the composite surveillance prioritization index. (**A**) Moran scatter plot showing significant positive global spatial autocorrelation (Global Moran’s I = 0.642, *p* < 0.001) using Queen contiguity spatial weights. The red line in the Moran scatter plot represents the fitted relationship between the district-level index and its spatial lag. (**B**) LISA cluster map based on 999 permutations and *p* < 0.05. High–High (HH) clusters included 120 districts, while Low–Low (LL) clusters included 57 districts. CRS: WGS 84/UTM Zone 47N (EPSG:32647).

The Global Moran’s I for $R_{p90}$ = 0.642 (*p* < 0.001), indicating significant positive spatial autocorrelation. High-priority districts were more likely to be near other high-priority districts, and low-priority districts were more likely to be near other low-priority districts. LISA identified 120 High–High (HH) districts and 57 Low–Low (LL) districts (Figure 3), a pattern consistent with the two broad high-priority corridors shown in Figure 2.

### 3.4. Final Shortlist of Priority Districts

**Figure 4:**
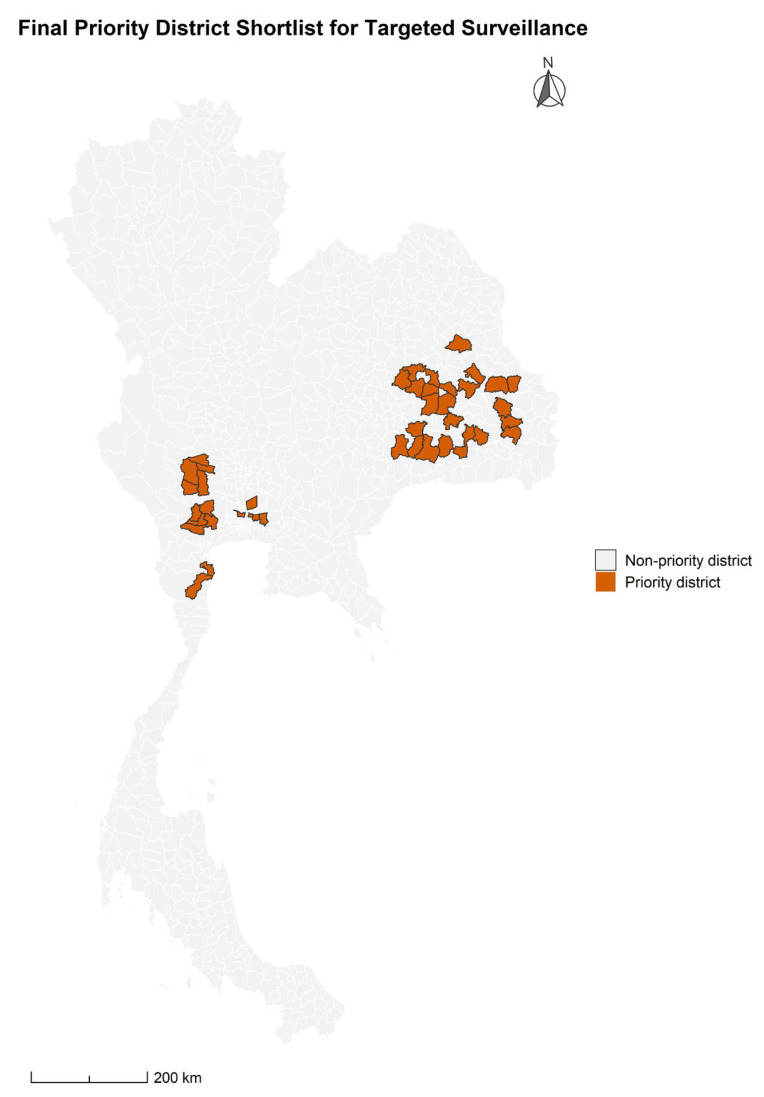
Final shortlist of priority districts for targeted surveillance. Orange polygons represent districts that met all three shortlisting criteria: significant High–High (HH) LISA classification (*p* < 0.05, Queen contiguity, 999 permutations), *R*p90 within the top 15% nationally, and total livestock population greater than or equal to the provincial 75th percentile. Gray polygons represent districts that did not meet the combined shortlisting criteria. A total of 42 districts were identified as priority surveillance districts. CRS: WGS 84/UTM Zone 47N (EPSG:32647). Data sources: DLD-certified slaughterhouse data and the official 2025 livestock census.

Thus, the final shortlist of districts for a targeted surveillance prioritization approach should at least comprise those that meet all the thresholds below: (1) LISA significant HH; (2) $R_{p90}$ within the top 15% nationally; (3) livestock population ≥ provincial P75. Thus, of the 120 significant HH districts, all those that passed the above criteria were further filtered to a shortlist of 42 priority districts (Figure 4). The top 20 districts for $R_{p90}$ are in Table 2 and were mainly located within the two broad high-priority corridors. The 42 priority districts were grouped into seven proposed operational zones using k-means clustering (*k* = 7, seed = 123) based on geographic proximity. Zone composition ranged from 2 to 11 districts, with median *R*p90 values ranging from 1.57 (Zone 1) to 3.30 (Zone 2), as detailed in Supplementary Table S1.

### 3.5. Sensitivity to Spatial Weight Definition

An additional definition of the neighborhood was evaluated. KNN k from 4 to 12 was deemed appropriate, with Moran’s I being high and significant (*p* < 0.001) and HH counts monotonically increasing with k; however, the geography of core high-priority areas remained broadly stable (Table 3). Queen contiguity was used as the baseline neighborhood definition for all primary analyses, as it reflects natural geographic adjacency between districts. As a sensitivity test, KNN with *k* ranging from 4 to 12 was additionally evaluated. Results were consistent across all values of *k* tested, with Moran’s I remaining high and statistically significant (*p* < 0.001), and the geographic extent of core high-priority areas remained stable (Table 3), supporting the robustness of the baseline Queen contiguity results.

### 3.6. Sensitivity to Model Parameters and Bootstrap Stability

Using the baseline shortlist 42 districts, we compared the baseline scenario (α=0.5, h=10  km) against four alternative scenarios using Jaccard similarity with the baseline shortlist. Results indicated high stability to spatial scale and moderate sensitivity to layer weights (Table 4). Bootstrap resampling (*N* = 200 iterations, Queen contiguity, set.seed = 123) further assessed the stability of the HH classification. Of the 42 shortlisted districts, 30 (71.4%) maintained HH status in 100% of iterations, and 31 (73.8%) showed stability of 90% or greater (median stability proportion = 1.00; mean = 0.78). At the national level, the proportion of HH-classified districts remained consistent across iterations (mean = 13.8%, SD = 0.24%), confirming that the overall spatial pattern was not driven by sampling variation. Nine shortlisted districts showed lower bootstrap stability (below 0.50), indicating marginal HH classification that warrants additional caution in operational planning.

## 4. Discussion

### 4.1. Spatial Heterogeneity of Slaughterhouses and Livestock Distribution

This study provides a national-scale spatial-epidemiology framework for the surveillance of livestock in Thailand. The spatial integration of slaughterhouse density and livestock population density identifies districts where the spatial overlap between slaughterhouse infrastructure and livestock populations may support prioritization of livestock health surveillance. Results of the study indicated that slaughterhouse and livestock populations were not equally distributed throughout the nation. Instead, slaughterhouses and livestock populations were concentrated in specific belts of the nation, specifically in northeastern and west-central Thailand. These findings help to frame slaughterhouses as infrastructure that is relevant to the livestock surveillance framework rather than as locations that are only relevant to the processing of those livestock populations. This finding is important because it suggests that uniform national surveillance allocation may be inefficient, and that surveillance resources should be concentrated where livestock populations and slaughterhouse infrastructure overlap.

These spatial patterns were determined based on the density of slaughterhouses derived from Gaussian KDE, and livestock census data at the district level, as is done with infrastructure and population-based surveillance prioritization [14,18,19,20]. The regional clustering was captured without smoothing out local patterns, as the fixed bandwidth of 10 km was used, and additional methodological developments in the future, such as space–time KDE, adaptive-bandwidth approaches, and modified spatial autocorrelation methods, could increase the sensitivity of these methods for future applications [21,22,23,24,25]. These results collectively reinforce the value of the combination of slaughterhouse facilities and animal numbers for planning of national-level surveillance [26,27,28].

### 4.2. National Composite Surveillance Prioritization Surface

Geographically, the composite surveillance prioritization surface of slaughterhouses and livestock population in Thailand identified the districts with priority areas. Each district has a value for *R*p90 ranging from −0.728 to 9.071 (median value of 0.063). These values are skewed to the right, indicating that the distribution of these values can be used to inform decisions for each district rather than utilizing the mean value of the national composite index [27,28]. Thus, the district-level *R*p90 values can be used to inform targeted surveillance planning, particularly when surveillance resources are limited and cannot be distributed uniformly across all districts. This supports the use of an upper-tail district summary such as *R*p90, because the main planning concern is not the average district condition but the presence of high-intensity areas that may require targeted surveillance attention.

### 4.3. Spatial Autocorrelation and Regional Clustering

At *p* < 0.001, the spatial autocorrelation test revealed a strong positive autocorrelation of the variable under assessment (Moran’s I = 0.642). LISA analysis indicated 120 significant High–High (HH) clusters and 57 Low–Low (LL) clusters at *p* < 0.05, reflecting the distinction between global and local spatial analyses of the variable under assessment [18,29]. The ability to recognize these HH and LL spatial clusters is useful for distinguishing areas, where surveillance intensification may be justified from areas where routine surveillance may be sufficient. Furthermore, the LISA analysis can be expanded in future studies to assess bivariate or multivariate data, such as the number of slaughterhouses in a district and the livestock density within each of those districts [30,31]. Future studies could also examine spatial instability by incorporating disease-rate data where available and by assessing heteroscedasticity in the spatial data [32].

### 4.4. Policy-Relevant Prioritization of High-Priority Districts

Significant HH clusters by LISA, the top 15% of *R*p90 values in the study area, and livestock population higher than the provincial P75 were the three criteria applied during the shortlisting process. Some districts were more strongly related to the slaughterhouse infrastructure, instead of the size of livestock populations, like some HH districts in Bangkok such as Sai Mai and Nong Chok, which have relatively low livestock populations. These districts, however, had high *R*p90 values, which suggests that they may be useful as infrastructure-based surveillance points in the registered slaughterhouse system [28,33]. These districts should therefore not be interpreted as livestock-production hotspots, but as infrastructure-driven surveillance priorities. The threshold and zoning choices should be interpreted as operational decision-support parameters rather than epidemiological cutoffs. In data-limited surveillance settings, pragmatic criteria are often needed to translate continuous spatial prioritization outputs into a field-ready shortlist. The top 15% threshold identifies the national upper tail of the index, while the provincial P75 criterion helps retain districts with locally important livestock populations. Similarly, the seven proposed operational zones provide a practical structure for field coordination among shortlisted districts, but they should not be interpreted as fixed administrative units or validated disease-risk zones. Future applications could refine these choices using animal movement data, slaughterhouse throughput, disease occurrence, biosecurity information, or formal stakeholder-defined resource constraints. The distinction is significant since livestock-dense districts may require strengthened population-based surveillance, while infrastructure-driven districts may require strengthened slaughterhouse inspection, data reporting, traceability, and coordination with the upstream supply chain. In addition, other slaughterhouse studies have suggested the potential gains from better infrastructure and capacity, especially when slaughterhouses have varying functions within the slaughtering, processing, and coordination of supply chains [34,35,36].

### 4.5. Model Robustness and Methodological Reliability

Several analyses were performed to assess the robustness of the main spatial pattern, across the range of model specifications. For the KNN sensitivity analysis (Table 3), the spatial size of core high-priority areas was broadly consistent with the Queen contiguity baseline, and HH classifications were consistently statistically significant for all the tested values of k. High shortlist overlap was also found for varying KDE bandwidths, and moderate overlap for varying layer weights was obtained from the parameter sensitivity analysis. Moreover, many districts kept their HH classifications when resampling with the bootstrap technique. Nine shortlisted districts showed bootstrap stability below 0.50, indicating marginal HH classification that should be interpreted with caution in field prioritization. This result indicates that the framework is more reliable for identifying the core high-priority pattern than for treating every shortlisted district as equally stable. Overall, the primary spatial pattern was less likely to be driven by a single model specification, but the exact composition of the shortlist should still be interpreted with attention to parameter sensitivity and bootstrap stability.

### 4.6. Broader Implications for One Health-Relevant Surveillance Planning

The main implication of this study contributes to a One Health-relevant surveillance planning framework by integrating registered slaughterhouse density and livestock population density to identify districts where surveillance resources may be prioritized [27,28,33,37,38]. Furthermore, the knowledge of the infrastructure of these slaughterhouses and the need for improvements to those facilities can lead to improved inspection of the livestock within those areas [34,35,36,39,40]. Additionally, the prioritization framework may also be modified to support the planning of future surveillance of zoonotic or livestock diseases, if relevant disease-specific data exist.

Although this study did not include human health or environmental datasets, the framework is One Health-relevant because slaughterhouses represent an operational interface between animal health surveillance, food safety inspection, zoonotic disease preparedness, and public health decision-making. Therefore, the term One Health-relevant is used to indicate policy relevance at the animal health–public health interface, rather than to imply that a full multi-sectoral One Health dataset was analyzed.

A key strength of this framework is that it uses routinely available administrative and surveillance-related data to generate an interpretable prioritization output for field planning. This is particularly relevant for data-limited settings where disease-specific data, movement records, or slaughterhouse throughput data may not be available at the national scale.

### 4.7. Limitations

**Figure 5:**
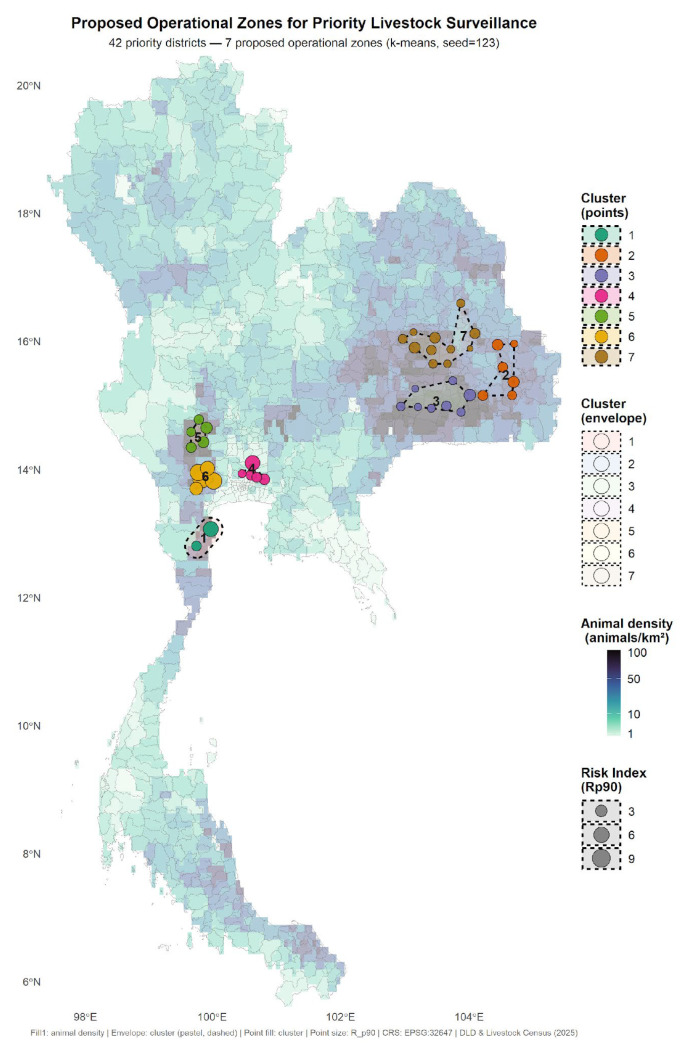
Proposed operational zones for the 42 priority surveillance districts. The 42 verified priority districts were grouped into seven proposed operational zones using k-means clustering (*k* = 7, seed = 123) based on district centroid coordinates. Point size is proportional to the composite surveillance prioritization index (*R*p90), and point color indicates operational zone assignment. Convex hull envelopes were used to visualize the geographic extent of each proposed zone. The background represents livestock density (animals/km^2^, log-scaled). These zones are intended to support field planning and coordinated surveillance implementation and should not be interpreted as disease-risk zones. CRS: WGS 84/UTM Zone 47N (EPSG:32647). Data sources: DLD-certified slaughterhouse data and the official 2025 livestock census.

Several limitations should be taken into account when interpreting the results. The first limitation of the framework is that it is based on district-level census data, making the assumption that the distribution of livestock is even within the district. This might overestimate the homogeneity of the sub-districts and could be explored in future research using more detailed information. Second, there was no standardization of the data across provinces; missing values were coded as zero, which may underestimate the priority in areas with low data density. Third, only legally registered slaughterhouses were included, and not the informal or unregistered slaughterhouses, which could have resulted in an underestimation of surveillance priority in areas where informal slaughterhouse activity is common. Fourth, the framework is a “snapshot” of the situation at a given time and does not include temporal considerations like seasonal livestock movements or disease incidence trends. Fifth, the equal weighting of slaughterhouse intensity and livestock density (α = 0.5) is a neutral assumption in this analysis, and results were moderately sensitive to this parameter (Jaccard = 0.644–0.711); future studies should consider data-driven weighting methods. Sixth, the index does not directly account for the number of pathogens detected, the risk of transmission, or the probability of an outbreak, due to the absence of pathogen, biosecurity, and animal movement data. In addition, human health and environmental datasets were not included; therefore, the framework should be interpreted as One Health-relevant rather than as a full One Health analysis. Seventh, the operational zones proposed in Figure 5 are the results of geographic clustering and are offered as a suggested planning framework for the field, not as analytic administrative zones. Eighth, the framework was designed and tested in Thailand and may need to be adapted for use in other countries with different data infrastructure, geographic, or regulatory characteristics.

## 5. Conclusions

This study developed a national spatial framework to prioritize livestock surveillance in Thailand based on registered slaughterhouse density and livestock population density at the district level. The framework identified 42 priority districts, grouped into seven proposed operational zones, mainly in the northeast and west-central parts of the country. These districts can be used as the initial basis for developing a targeted surveillance plan, prioritizing inspections and allocating resources using a One Health-relevant approach. The index is not a disease-risk indicator but should be used to prioritize surveillance, as it is not based on disease occurrence, pathogen detection, animal movement, slaughterhouse throughput, or biosecurity data. Potential future studies could combine the integration of movement and incidence data with the use of STKDE [21,22] and adaptive-bandwidth approaches [23], and the use of local tests such as SaTScan with LISA could improve the confirmation of high-priority spatial clusters [26]. In addition to methodological development, combining slaughterhouse-based surveillance with value-chain governance and operational planning could be used to further enhance the allocation of surveillance resources [33,34,35,36,39,40].

## Figures and Tables

**Table 1:** Descriptive statistics for the final district-level composite surveillance prioritization indices (*N* = 928).

Metric	Min	P25	Median	Mean	P75	P90	Max	SD
**Rmean**	−0.728	−0.434	−0.066	0.157	0.501	1.227	6.807	0.848
**Rp90**	−0.728	−0.339	0.063	0.308	0.655	1.420	9.071	0.971

**Table 2:** Top 20 priority districts ranked by *R*p90.

Rank	District	Province	Operational Zone	*R*p90	Total Animals
1	Ban Pong	Ratchaburi	6	9.07	38,544
2	Mueang Nakhon Pathom	Nakhon Pathom	6	7.25	17,670
3	Tha Maka	Kanchanaburi	6	6.94	43,628
4	Mueang Phetchaburi	Phetchaburi	1	5.71	54,580
5	Khlong Luang	Pathum Thani	4	5.45	1822
6	Kamphaeng Saen	Nakhon Pathom	6	4.90	33,235
7	Photharam	Ratchaburi	6	3.65	30,498
8	Don Chedi	Suphan Buri	5	2.95	47,511
9	Uthumphon Phisai	Si Sa Ket	3	2.92	52,293
10	Mueang Ubon Ratchathani	Ubon Ratchathani	2	2.87	41,398
11	Wapi Pathum	Maha Sarakham	7	2.62	82,282
12	U Thong	Suphan Buri	5	2.60	85,276
13	Mueang Amnat Charoen	Amnat Charoen	2	2.59	51,749
14	Sai Mai	Bangkok	4	2.51	92
15	Nong Chok	Bangkok	4	2.51	2517
16	Mueang Roi Et	Roi Et	7	2.47	35,828
17	Kut Chum	Yasothon	7	2.25	53,201
18	Huai Krachao	Kanchanaburi	5	2.23	62,489
19	Mueang Si Sa Ket	Si Sa Ket	2	2.19	48,940
20	Muang Sam Sip	Ubon Ratchathani	2	2.11	45,287

**Table 3:** Sensitivity of Global Moran’s I and HH counts to KNN k.

No. of Neighbors (*k*)	Global Moran’s I	*p*-Value	Number of HH Districts
4	0.677	<0.001	106
6	0.624	<0.001	120
8	0.570	<0.001	145
10	0.535	<0.001	157
12	0.504	<0.001	158

**Table 4:** Sensitivity of the operational shortlist to parameter changes.

Scenario	Alpha (α)	Bandwidth (km)	No. of Shortlisted Districts	Jaccard Similarity with Baseline
Baseline	0.5	10	42	1
Alpha = 0.7 (Slaughterhouse focus)	0.7	10	34	0.644
Alpha = 0.3 (Livestock focus)	0.3	10	37	0.711
Bandwidth = 5 km (Narrow view)	0.5	5	32	0.800
Bandwidth = 20 km (Wide view)	0.5	20	51	0.784

## Data Availability

The data presented in this study are available on request from the corresponding author. Certified slaughterhouse location data were provided directly by Thailand’s Department of Livestock Development (DLD), Bureau of System Development and Livestock Product Standard Certification; raw coordinates are not publicly redistributable. Information on the responsible DLD bureau is available at https://certify.dld.go.th/index.php?lang=th (accessed on 1 June 2026). Derived outputs, including raster surfaces and district-level summaries, are available from the corresponding author upon reasonable request. Livestock population data were obtained from public reports of the Department of Livestock Development, Information and Communication Technology Center, available at https://ict.dld.go.th/index.php/th/service/report (accessed on 1 June 2026). Analytical code is available from the corresponding author upon reasonable request.

## Supplementary Materials

The following supporting information can be downloaded at https://www.mdpi.com/article/10.3390/ani16121767/s1, Table S1: All 42 priority districts for targeted livestock surveillance, ranked by composite surveillance prioritization index (*R*p90).

## Abbreviations

The following abbreviations are used in this manuscript:

FMD	Foot-and-mouth disease
KDE	Kernel density estimation
LISA	Local Indicators of Spatial Association
LSD	Lumpy skin disease
