# Supplementary material for: A Slaughterhouse-Linked Spatial Framework for Prioritizing Livestock Surveillance in Thailand
Source: Animals (Basel). 2026 Jun 8;16(12):1767. doi: 10.3390/ani16121767 (PMC13295695; doi:10.3390/ani16121767)
Supplement: Supplementary file 1 [file animals-16-01767-s001.zip › animals-4340817-supplementary.pdf]

Supplementary Table S1. All 42 priority districts for targeted livestock surveillance, ranked by composite surveillance prioritization index (Rp90).

Districts meeting all three shortlisting criteria: (1) significant High-High (HH) LISA cluster ($p < 0.05$, Queen contiguity, 999 permutations); (2) Rp90 within the top 15% nationally; and (3) total livestock population greater than or equal to the provincial 75th percentile. Rp90 = 90th percentile of the composite surveillance prioritization index. Total Animals = district-level total of beef cattle, dairy cattle, and buffalo. Zone = proposed operational zone assignment based on k-means geographic clustering (k=7, seed=123).

Rank	District	Province	Operational Zone	*Rp90*	Total Animals
1	Ban Pong	Ratchaburi	6	9.07	38,544
2	Mueang Nakhon Pathom	Nakhon Pathom	6	7.25	17,670
3	Tha Maka	Kanchanaburi	6	6.94	43,628
4	Mueang Phetchaburi	Phetchaburi	1	5.71	54,580
5	Khlong Luang	Pathum Thani	4	5.45	1,822
6	Kamphaeng Saen	Nakhon Pathom	6	4.90	33,235
7	Photharam	Ratchaburi	6	3.65	30,498
8	Don Chedi	Suphan Buri	5	2.95	47,511
9	Uthumphon Phisai	Si Sa Ket	3	2.92	52,293
10	Mueang Ubon Ratchathani	Ubon Ratchathani	2	2.87	41,398
11	Wapi Pathum	Maha Sarakham	7	2.62	82,282
12	U Thong	Suphan Buri	5	2.60	85,276
13	Mueang Amnat Charoen	Amnat Charoen	2	2.59	51,749
14	Sai Mai	Bangkok	4	2.51	92
15	Nong Chok	Bangkok	4	2.51	2,517
16	Mueang Roi Et	Roi Et	7	2.47	35,828
17	Kut Chum	Yasothon	7	2.25	53,201
18	Huai Krachao	Kanchanaburi	5	2.23	62,489
19	Mueang Si Sa Ket	Si Sa Ket	2	2.19	48,940
20	Muang Sam Sip	Ubon Ratchathani	2	2.11	45,287
21	Khlong Sam Wa	Bangkok	4	2.05	1,048
22	Tha Yang	Phetchaburi	1	2.00	44,011
23	Sikhoraphum	Surin	3	1.91	86,582
24	Nong Ya Sai	Suphan Buri	5	1.90	37,681

Rank	District	Province	Operational Zone	*Rp90*	Total Animals
25	Chaturaphak Phiman	Roi Et	7	1.89	46,214
26	Warin Chamrap	Ubon Ratchathani	2	1.76	39,641
27	Lao Khwan	Kanchanaburi	5	1.75	98,687
28	Borabue	Maha Sarakham	7	1.68	66,165
29	Kaset Wisai	Roi Et	7	1.64	78,501
30	Mueang Buri Ram	Buri Ram	3	1.61	88,247
31	Kuchinarai	Kalasin	7	1.57	25,433
32	Prang Ku	Si Sa Ket	3	1.53	39,103
33	Rattanaburi	Surin	3	1.50	54,427
34	Pak Kret	Nonthaburi	4	1.49	548
35	Mueang Surin	Surin	3	1.46	104,605
36	At Samat	Roi Et	7	1.42	30,525
37	Suwannaphum	Roi Et	7	1.35	73,458
38	Pathum Ratchawongsa	Amnat Charoen	2	1.31	20,357
39	Krasang	Buri Ram	3	1.28	62,847
40	Satuek	Buri Ram	3	1.25	52,468
41	Mueang Maha Sarakham	Maha Sarakham	7	1.24	31,221
42	Mueang Yasothon	Yasothon	7	1.16	27,017
